# *Raynals*, an online tool for the analysis of dynamic light scattering

**DOI:** 10.1107/S2059798323004862

**Published:** 2023-07-10

**Authors:** Osvaldo Burastero, George Draper-Barr, Bertrand Raynal, Maelenn Chevreuil, Patrick England, Maria Garcia Alai

**Affiliations:** a European Molecular Biology Laboratory, Notkestrasse 85, Hamburg, Germany; b Centre for Structural Systems Biology, Notkestrasse 85, 22607 Hamburg, Germany; c Institut Pasteur, Université Paris Cité, CNRS UMR 3528, Plateforme de Biophysique Moléculaire, Paris, France; National Centre for Biological Sciences-TIFR, India

**Keywords:** *Raynals*, dynamic light scattering, molecular biophysics, online data analysis, protein quality control, hydrodynamic radius, size distribution, software

## Abstract

Dynamic light scattering (DLS) is routinely employed to assess the homogeneity and size-distribution profile of samples containing microscopic particles in suspension or solubilized polymers. In this work, *Raynals*, user-friendly software for the analysis of single-angle DLS data that uses the Tikhonov–Phillips regularization, is introduced. Its performance is evaluated on simulated and experimental data generated by different DLS instruments for several proteins and gold nanoparticles. DLS data can easily be misinterpreted and the simulation tools available in *Raynals* allow the limitations of the measurement and its resolution to be understood. It was designed as a tool to address the quality control of biological samples during sample preparation and optimization and it helps in the detection of aggregates, showing the influence of large particles. Lastly, *Raynals* provides flexibility in the way that the data are presented, allows the export of publication-quality figures, is free for academic use and can be accessed online on the eSPC data-analysis platform at https://spc.embl-hamburg.de/.

## Abstract 2

*Raynals* is an online, user-friendly, free (for academia) and advanced tool for the analysis of single-angle dynamic light-scattering data. Estimation of the size distribution is performed through the Tikhonov–Phillips regularization.

## 1. Introduction

The polarizability of light by biological samples is not homogeneous due to macromolecules in suspension undergoing Brownian motion; an incident light beam therefore scatters in all directions and the scattering intensity fluctuates over time. From the average intensity of the scattered light, valuable information about the molecular weight, the radius of gyration, the internal spatial arrangement of scattering centres and the virial coefficients can be obtained (Schmitz, 1990 ▸). Moreover, the measured macroscopic intensity fluctuation of the scattered light (not the fluctuations in intensity from individual molecules) allows the estimation of the apparent translational diffusion coefficient *D*_app_ and the related hydrodynamic radius *R*_h_ (Schmitz, 1990 ▸).

Dynamic light-scattering (DLS) experiments consist of illuminating a sample with a polarized monochromatic laser and detecting the scattered light with a high temporal resolution. The diffracted light undergoes constructive or destructive interference by the surrounding particles, generating an intensity fluctuation that correlates to the timescale movements of the particles. This dynamic information on the scattering by the particles is collected after passage through a second polarizer. The second-order autocorrelation function is then constructed from the acquired intensity trace to determine *D*_app_. Samples that can be fitted using a single exponential decay are considered to be monodisperse. In addition, polydisperse systems require a sum of exponential decays. *D*_app_ can be estimated at single or multiple angles, with the angular dependence of the signal revealing the shape of the scattering particle.

DLS is a fast, nondestructive and low-consumption method that is routinely used to assess the homogeneity and aggregation state of protein samples (Raynal *et al.*, 2014 ▸; de Marco *et al.*, 2021 ▸; Stetefeld *et al.*, 2016 ▸). It can also be used for more advanced applications such as the calculation of the critical micelle concentration (CMC) of detergents (Sutherland *et al.*, 2009 ▸), the optimization of solutions for protein–detergent complexes (Meyer *et al.*, 2015 ▸) and the study of protein crystallization (Saridakis *et al.*, 2002 ▸; Dierks *et al.*, 2008 ▸; Meyer *et al.*, 2012 ▸; Oberthuer *et al.*, 2012 ▸; Schubert *et al.*, 2017 ▸). A limitation of this technique is that the scattering signal is extremely sensitive to the particle ratio, so large aggregates overshadow the signal from smaller particles, even if the latter population is a greater proportion of the total particle number in solution. In addition, deconvolution of the correlogram is an ill-posed problem, implying that it is not possible to retrieve the original intensity-weighted particle-size distribution. For single-angle measurements, the rule of thumb is that only species that differ by a factor of two or three in their *R*_h_ can be totally distinguished, and technical instrumentation limitations expand this factor to a factor of five. Finally, the transformation of the intensity distribution to mass (or volume) distribution is subject to additional assumptions such as the assumption that particles are perfect hard spheres with constant density.

Results from DLS experiments are typically analysed using the commercial software provided by the instrument vendor. Occasionally, advanced users decide to fit their data using desktop programs such as *CONTIN* or *SEDFIT* (Provencher, 1982 ▸; Brown *et al.*, 2007 ▸). Here, we introduce *Raynals*, an online tool designed for the interpretation of DLS data tailored to biological samples. *Raynals* is our newest addition to eSPC (https://spc.embl-hamburg.de/), an online data-analysis platform that contains, so far, modules for evaluating experimental data from differential scanning fluorimetry, mass photometry and microscale thermophoresis (Burastero *et al.*, 2021 ▸; Niebling *et al.*, 2021 ▸, 2022 ▸).

## 2. Methods

### 2.1. Input data

The final measurement provided by a DLS experiment is the intensity correlation function (or second-order correlation function). This function, called *G*_2_(τ), can be expressed as an integral over the product of the intensities at time *t* and a delayed time *t* + τ,

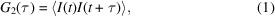

where τ is the lag time between two time points. Generally, DLS instruments export the normalized version of G_2_(τ), 

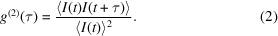

The normalized second-order autocorrelation function g^(2)^(τ) is the input data for *Raynals*.

### 2.2. Fitting DLS data

#### 2.2.1. Theory

The normalized second-order autocorrelation function g^(2)^(τ) can be related to the normalized first-order correlation function *g* ^(1)^(τ) through the Siegert equation (Siegert, 1943 ▸),

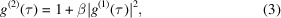

where β is the coherence factor, which depends on the instrument and on the scattering properties of the macromolecules. The function *g* ^(1)^(τ) contains information about the motion of the particles, and for monodisperse samples it decays exponentially according to one decay constant. On the other hand, for polydisperse systems *g* ^(1)^(τ) is represented by an intensity-weighted integral over a distribution of decay rates *G*(Γ) (equation 13). Each decay rate is associated with a certain diffusion coefficient according to

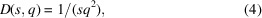

where *s* is the inverse of the decay rate and *q* is the Bragg wavevector defined as

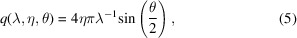

where λ, η and θ are the wavelength of the incident light, the refractive index of the solvent and the angle of detection, respectively. Finally, the diffusion factors (*D*) can be transformed to hydrodynamic radii (*R*_h_) with the Stokes–Einstein relation

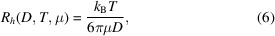

where *T* and μ are the temperature and viscosity, respectively, and *k*_B_ is the Boltzmann constant.

#### 2.2.2. Fitting algorithm (Tikhonov–Phillips regularized inversion)

*Raynals* fits the first-order autocorrelation data based on the so-called Tikhonov–Phillips regularized inversion. For this purpose, we first obtain β by fitting a second-degree polynomial to the DLS data at times shorter than 5 µs. We then apply equation (3) to calculate *g*_1_(τ). Due to the square root in this equation, *g* ^(1)^(τ) can only be computed when *g* ^(2)^(τ) ≥ 1. Therefore, we only evaluate the data before the first occurrence of *g* ^(2)^(τ) < 1.

After calculating *g* ^(1)^(τ), we discretize the decay-rate space by using *n* (*n* = 200) points between 0.1 and 10^6^ nm log spaced on the hydrodynamic radius scale.

The equation that we need to fit becomes

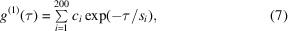

subject to the constraints

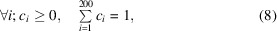




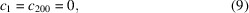

where *c_i_* is the *i*th contribution of the *i*th inverse decay rate (*s_i_*). Due to the ill-conditioned nature of the problem (infinite possible solutions), we need to add a regularization term, so we simultaneously solve the following equations,

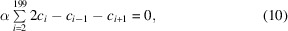

where α is a regularization parameter controlling how close the relative contribution of each (inverse) decay rate should be to its neighbouring (inverse) decay rates. The whole set of linear equations is solved together using the non-negative least-squares solver from the *SciPy* package (https://docs.scipy.org/doc/scipy/reference/generated/scipy.optimize.nnls.html).

#### 2.2.3. Implementation of the *L*-curve criterion

To build the *L*-curve, a sequence of regularization parameters (α) evenly spaced on a log scale are evaluated. This sequence is generated using the formula

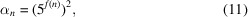

where *f*(*n*) depends on three parameters called ‘start’, ‘stop’ and ‘step’, and is defined as

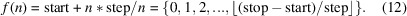

The corner of the curve is then detected by applying the triangle method (Castellanos *et al.*, 2002 ▸).

### 2.3. Simulated DLS data

#### 2.3.1. Generation protocol

All of the artificially generated data are based on the light scattered by populations of particles that have a normal distribution in the hydrodynamic radius space. The necessary steps are as follows.

(i) Obtain a sample of particles with a certain normal hydrodynamic radius distribution.
(ii) Compute the intensity of the light scattered by each particle using the Mie theory (implemented in the *Miepython* package, version 2.3.1; Prahl, 2023 ▸). This step requires the selection of an angle of detection, a laser wavelength and a refractive index.
(iii) Discretize the hydrodynamic radius space using a log scale from 0.1 to 10^6^ nm and calculate the amount of scattered light in each interval.
(iv) Divide the scattered light in each interval by the total scattered light to obtain the relative contributions.
(v) Convert each hydrodynamic radius into diffusion coefficients at a certain temperature and viscosity.
(vi) Apply equations (4), (7) and (3) to calculate the final autocorrelation curve.
(vii) Add uncorrelated normally distributed error to the autocorrelation curves.

For all of the simulations, the wavelength, detection angle, temperature, refractive index and viscosity were set to 817 nm, 150°, 1.33 and 0.00089 Pa s, respectively. The parameter β (from equation 3) and the standard deviation of the normally distributed error were set to 0.2 and 0.002, respectively.

#### 2.3.2. Analysis protocol

For the fitting, the same values of refractive index and viscosity were used. The ‘start’, ‘stop’ and ‘step’ values to build the *L*-curves were −6, 1 and 0.2, respectively. The region of interest to estimate the sample *R*_h_ was 0.1–100 nm.

### 2.4. Experimental samples

#### 2.4.1. Commercial samples

Carbonic anhydrase (CA) from bovine erythrocytes (CAS 9001-03-0), monomeric bovine albumin (BSA; CAS 9048-46-8) and gold nanoparticles [GP; Product Nos. 741949 (radius of 2.5 nm), 741965 (radius of 10 nm) and 741981 (radius of 20 nm)] were purchased from Sigma–Aldrich. CA and BSA were dissolved in phosphate-buffered saline pH 7.4.

#### 2.4.2. In-house-produced samples

The recombinant expression of a β-propeller domain (BPD), an intrinsically disordered protein (IDP) and a coiled-coil dimeric polypeptide (CC) was performed according to the following protocol (protein sequences are provided in the supporting information). *Escherichia coli* BL21 Gold (DE3) cells containing the pLysS plasmid were transformed with pETM30 plasmids containing the respective cDNAs for the globular and coiled-coil polypeptides with an N-terminally fused His_6_-TEV cleavage site–glutathione *S*-transferase (GST) affinity-purification tag. The cells were grown in 2×YT medium to an OD of 0.6 before induction with 0.2 m*M* isopropyl β-d-1-thiogalactopyranoside and left shaking at 20°C overnight. The cultures were harvested at 7000 rev min^−1^ for 20 min before resuspension in lysis buffer (30 m*M* Tris pH 8, 200 m*M* NaCl, 5% glycerol). The cells were passed through an Emulsiflex several times and the cell debris was sedimented at 38 000*g* for 1 h at 4°C. The proteins were purified using immobilized metal-affinity chromatography (IMAC). After removal of the purification tag by TEV protease and a second round of IMAC, the target proteins were further purified by gel filtration (Superdex 200 Increase 10/300 GL) in SEC buffer (30 m*M* Tris pH 8, 150 m*M* NaCl, 1 m*M* DTT). For IDP, we used the same protocol but with a C-terminal His_6_ tag in a pnEA vector transformed into *E. coli* BL21 (DE3) cells without any additional plasmids.

### 2.5. Dynamic light-scattering experiments

Dynamic light-scattering (DLS) experiments for CA, BSA and GP were carried out using a Wyatt DynaPro Plate Reader III instrument (wavelength of 817 nm and angle of detection of 150°). CA and BSA were centrifuged at 17 000 rev min^−1^ for 15 min before measurement (the GP were not centrifuged). DLS experiments for BPD, IDP and CC were performed using a Wyatt DynaPro NanoStar (cuvette holder, wavelength of 658 nm and angle of detection of 90°). The globular domain, IDP and CC were centrifuged at 21 000*g* for 20 min before measurement. The temperature was set to 20°C. The running parameters used to obtain the DLS curves are described in Table 1 ▸.

For the fitting of all samples, the viscosity (0.00089 P s) and refractive index (1.33) of water were used. The ‘start’, ‘stop’ and ‘step’ values used to build the *L*-curves were −6, 2 and 0.25, respectively.

### 2.6. *AlphaFold* models

*AlphaFold* structures of BPD, CC and IDP was obtained with *ColabFold* version 1.5.2: *AlphaFold*2 using *MMseqs*2 (Mirdita *et al.*, 2022 ▸). For CC, we predicted the homodimer. Default parameters were used for all proteins.

### 2.7. *R*_h_ prediction

The *R*_h_ values were predicted using the online tool provided by Fluidic Analytics available at https://www.fluidic.com/toolkit/hydrodynamic-radius-converter. The corresponding molecular weights were 40, 107 and 65 kDa for BDP, CC (homodimer) and IDP, respectively.

## 3. Results and discussion

### 3.1. *Raynals* workflow

**Figure 1:**
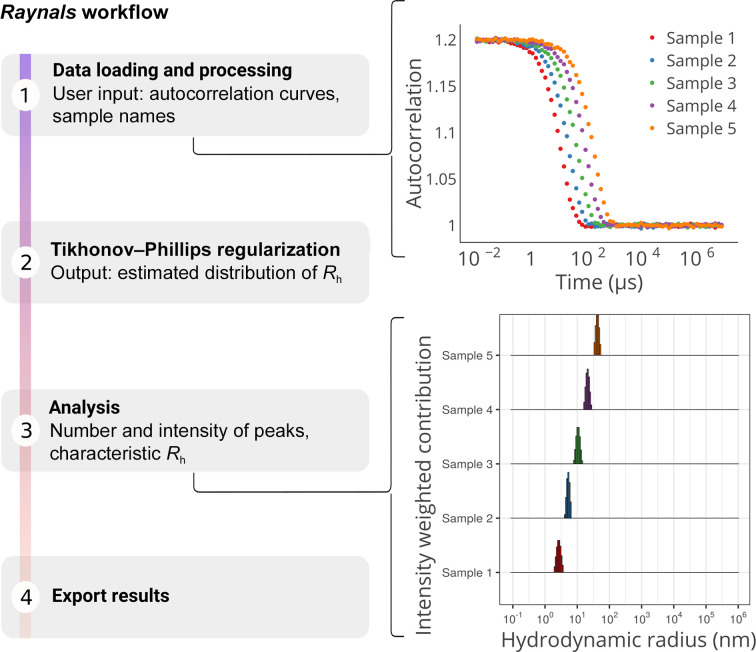
The *Raynals* pipeline has four steps. Firstly, the second-order autocorrelation data are loaded and filtered based on initial value and baseline quality. Input parameters include the detection angle, laser wavelength, temperature, refractive index and viscosity. Secondly, a regularization approach is used to fit the first-order autocorrelation data, assuming a smooth nonparametric distribution of decay rates (or hydrodynamic radii). Thirdly, a threshold based on residuals can be used to remove poorly fitted curves and the estimated distributions are displayed. The user must select regions of interest to extract information about the peaks (for example the contribution to the total intensity). Finally, the user can export the *R*_h_ distribution and the associated second-order autocorrelation curves.

The workflow for the use of the *Raynals* tool can be divided into four steps (Fig. 1 ▸). To begin with, the user loads the normalized second-order autocorrelation curves and the associated experimental information, such as the angle of detection and the laser wavelength. Raw curves can be filtered by removing those with a lower intercept or a ‘bumpy’ baseline to exclude samples with aggregates and/or buffers (see user documentation: https://spc.embl-hamburg.de/assets/apps_user_documentation/RaynalsUserDocumentation.pdf, Filtering section). Sample preparation by centrifugation and filtration is a critical step to remove dust particles and aggregates from the solution that would introduce artefacts into the measurements.

DLS data are fitted using models based on the Siegert relationship, which connects the normalized second-order *g* ^(2)^(τ) and first-order *g* ^(1)^(τ) autocorrelation functions. *g* ^(1)^(τ) can be represented by an intensity-weighted integral over a distribution of decay rates *G*(Γ) (Xu, 2006 ▸),

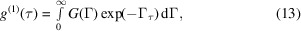

where *G*(Γ) is normalized as follows:

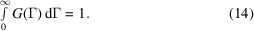




Finding the distribution *G*(Γ) from noisy data is challenging, and there are several available methods that can be used (Schmitz, 1990 ▸). One common approach is to use the cumulants method proposed by Koppel to estimate the mean and variance of the distribution (Koppel, 1972 ▸). These values are used to calculate the polydispersity index (PdI) and the percentage polydispersity (%PdI):

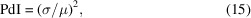




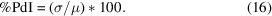

In this method, μ and σ are the estimated sample mean and standard deviation, respectively. The formula for %PdI is exactly the same as that for the percentage of coefficient of variation (%CV). It has been stated that values of PdI below 0.05 (%PdI < 22) correspond to monodisperse colloidal particles and values close to unity (or above) indicate polydisperse samples. Despite being recommended by the International Organization for Standardization (ISO 13321 and ISO 22412), the cumulants method is highly sensitive to small amounts of aggregates and may yield misleading results in non-monodisperse samples (Mailer *et al.*, 2015 ▸).

Alternative approaches for determining *G*(Γ) include adjusting a discrete number of exponentials with different decay rates that do or do not follow a parametric distribution. Two known methods are the non-negative least-squares (NNLS) method and the exponential sampling method (Morrison *et al.*, 1985 ▸; Ostrowsky *et al.*, 1981 ▸). In *Raynals*, we implemented the fitting of *g* ^(1)^(τ) through the Tikhonov–Phillips regularized inversion, which is commonly used to solve ill-posed inverse problems (Phillips, 1962 ▸; Provencher, 1982 ▸; Brown *et al.*, 2007 ▸). This method requires the selection of a regularization matrix (*L*) and a regularization parameter (α), and then finding the vector of relative contributions (*x*) such that

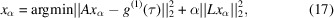

where *A* is the kernel matrix with values *a*_*i*,*j*_,

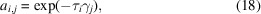

where *i* and *j* iterate over the lag-time and decay-rate vectors, respectively. In *Raynals*, the decay-rate space is discretized in such a way that the hydrodynamic radius points are evenly spaced on a log scale, and *L* is the second-order derivative matrix that constrains how close the value of each decay rate is from its neighbours.

Thirdly, the distribution of decay rates is transformed into a distribution of diffusion coefficients and subsequently into a distribution of *R*_h_ (Stokes–Einstein relation, equation 6). The fitted curves can then be filtered based on the residuals. To report the *R*_h_ (or diffusion coefficients), we apply a peak-searching algorithm within user-selected intervals (for example 1–100 nm). In the following sections, we assess the performance of the developed software, highlighting its capabilities and limitations after analysing experimental and simulated data.

### 3.2. Addressing the performance of *Raynals* with simulated DLS data

**Figure 2:**
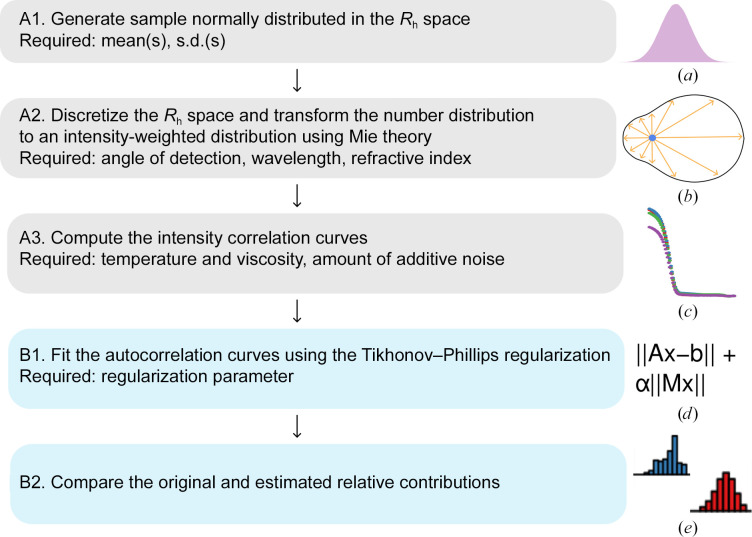
Workflow of the simulations performed to evaluate the capacity of Tikhonov–Phillips regularization to return the original hydrodynamic radius distribution. Grey and blue boxes represent the data-generation and data-fitting steps, respectively. Figures on the right from top to bottom: (*a*) normal distribution, (*b*) light scattered by a particle at different angles according to the Mie theory, (*c*) simulated autocorrelation curves, (*d*) equation of the regularization approach required to solve a nonlinear inverse problem and (*e*) histograms of the fitted intensity distributions.

DLS data have been simulated based on the following protocol (Fig. 2 ▸). To start with, we created samples with a number-weighted Gaussian distribution of *R*_h_. Then, using the Mie theory (van de Hulst, 1981 ▸; Mie, 1908 ▸), we calculated the scattered light intensity of the particles, assuming that they were perfect spheres. Finally, we discretized the *R*_h_ space, computed the relative contributions to the total intensity of each interval and obtained the autocorrelation curves. It is important to note that both the intensity and number distribution are just different physical representations of the same reality. All data were generated using the ‘Simulation’ panel in *Raynals*. Explanatory files containing the parameters used to reproduce the simulations are available to download, see Section 6.

#### 3.2.1. Case 1. One population, low %CV

To test the simplest case, we simulated the autocorrelation curves of a monodisperse sample (defined by %CV = 10%). It has been reported that using the weighted harmonic mean (WHM) to estimate the particle size would be a better alternative than the weighted mean (WM) because it matches the average size from the cumulants analysis for samples with low PdI (Farkas & Kramar, 2021 ▸). For data sets containing non-negative values, the harmonic mean is lower than or equal to the geometric mean, and the geometric mean is lower than or equal to the arithmetic mean (Bullen, 2003 ▸). In our analysis, we have compared the usage of the WHM or the highest peak value from the distribution (mode) for obtaining *R*_h_. The fitted distributions are available for download in *Raynals* and users can choose their preferred *R*_h_ reporting method.

**Figure 3:**
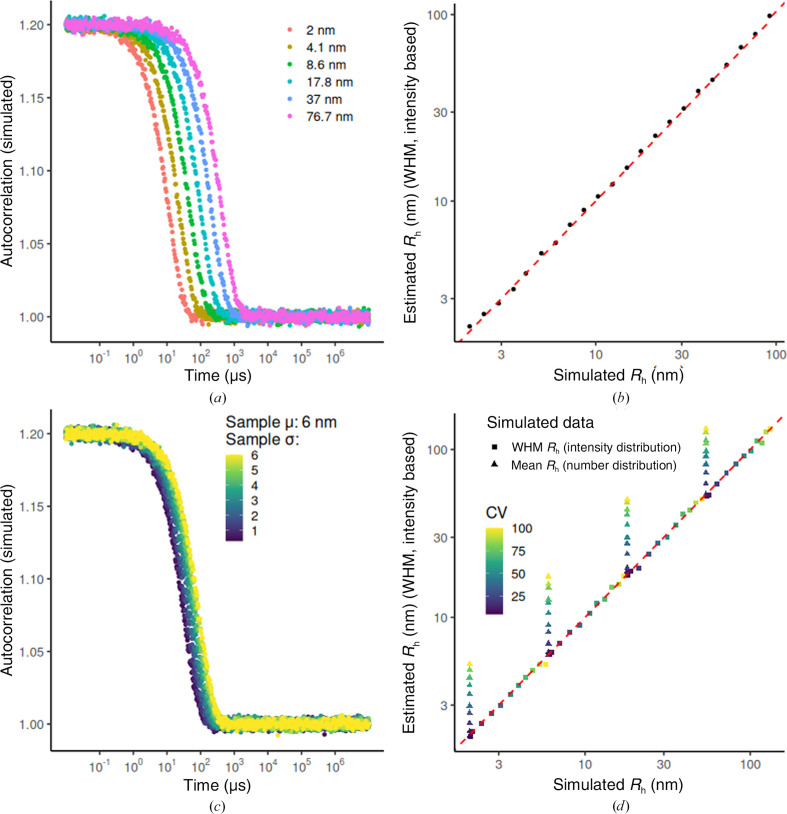
(*a*) Simulated autocorrelation curves for samples following a number-weighted normal distribution with a %CV of 10% (for example a mean of 2 nm and standard deviation of 0.2 nm). (*b*) Estimated hydrodynamic radius (*R*_h_) (WHM, intensity-based) versus the mean *R*_h_ from the simulated number-based distributions. (*c*) Example curves of the generated samples following a number-weighted normal distribution with a mean of 6 nm and %CVs ranging from 5% to 100%. (*d*) Estimated *R*_h_ (WHM, intensity-based) versus the WHM and mean *R*_h_ from the simulated intensity-based and number-based distributions, respectively. In (*b*) and (*d*) the red line indicates a perfect fitting. To estimate the WHM an arbitrary α of 0.01 was used.

Our results show that the estimated and original *R*_h_ values are in excellent agreement (Fig. 3 ▸), with the mean *R*_h_ from the number distribution remaining constant after transforming to an intensity-weighted distribution (Supplementary Fig. S1*a* ). Consistently, we observed no significant differences between using the peak maximum or the weighted harmonic mean (WHM) to estimate *R*_h_ (Supplementary Fig. S1*b* ).

#### 3.2.2. Case 2. One population, increased %CV

To further evaluate the efficacy of the Tikhonov–Phillips regularization method with a second-order derivative matrix as a penalization term, we fitted 44 samples (μ = 2, 6, 18 or 54 nm) with %CVs ranging from 5% to 100% (Fig. 3 ▸ *c*). The resulting WHM based on the fitted intensity distribution consistently correlated with the WHM derived from the simulated intensity distribution (Fig. 3 ▸ *d*, squares). However, for the samples with higher %CVs (>25%), the estimated WHM *R*_h_ did not agree with the mean *R*_h_ from the simulated number distribution (Fig. 3 ▸ *d*, triangles). As expected, these simulations highlight how the algorithm can effectively retrieve the correct *R*_h_ from the underlying intensity distribution, but it may not be as accurate when compared with the number distribution (Fig. 3 ▸ *d*, triangles).

It would be highly beneficial to determine whether we can recover the original particle-size distribution in addition to the ‘characteristic’ *R*_h_. In this sense, the regularization parameter α is crucial and the solution may be completely under-smoothed or over-smoothed. When the amount of noise is unknown, the value of α is typically selected through an *a posteriori* empirical rule. To date, there is no agreed-upon criterion on how to select the abovementioned rule. Some applied criteria are the *L*-curve (Lawson & Hanson, 1974 ▸), the *U*-curve (Krawczyk-Stańdo & Rudnicki, 2007 ▸), the composite residual and smooth operator (CRESO; Cheng *et al.*, 2003 ▸), the product (Lian *et al.*, 1998 ▸), the zero-crossing (Cheng *et al.*, 2003 ▸) and the general-cross validation (Hansen, 1994 ▸) methods. Appropriate evaluation of the various available methods remains an active area in DLS research. Two questions arise naturally: (i) can we use a fixed α for comparing the sample width? and (ii) is there a way to find the optimal value of α that produces an estimated distribution similar to the original distribution? The goal is to find a correlation between the ‘true standard deviation’ (a proxy for polydispersity) from the generated and the estimated distributions. To address the first question, we analysed the created samples using different values of α (0.0001, 0.001, 0.01, 0.1 and 1).

Table 2 ▸ shows the correlation values between the estimated and true standard deviation values for the simulated distributions (Supplementary Fig. S2). These results suggest that for one population with the given level of noise, the proposed fitting method is useful for comparing standard deviations. An example of fitted versus simulated intensity distributions is provided in Supplementary Fig. S3. The value of α that gives the closest distribution on average is α = 1 (Supplementary Fig. S4). However, this value (α = 1) fails when used to determine the distribution of samples with %CV equal to 5% due to an over-smoothing effect (Supplementary Fig. S4).

To improve the accuracy of the sample-distribution estimation, we evaluated the efficacy of the *L*-curve criteria. It has previously been shown that this method yields correct particle-size distributions for DLS data from microgel suspensions (Scotti *et al.*, 2015 ▸). The corresponding heuristic rule consists of plotting the logarithm of the residuals (fidelity term) against the logarithm of the norm of the regularized solution (penalty term) for different values of α and selecting the value of α that corresponds to the corner point of the *L*-shaped curve (Supplementary Fig. S5). Hereby, we achieve a balance between the size of the regularized solution and the accuracy of the fit. To find the corner of the *L*-curve we used the triangle method proposed by Castellanos *et al.* (2002 ▸). In our simulations, this approach resulted in finding values of α which also gave a significant correlation between the expected and estimated standard deviations. However, the α values were sometimes suboptimal (Supplementary Fig. S4). Nonetheless, this strategy is better than arbitrarily selecting a fixed α, as seen for example by the distance between the estimated and true intensity distributions for α = 10^−4^. In *Raynals*, it is also possible to explore different values of regularization terms and export them together with the penalty (||*Lx*|| in equation 17) and fidelity terms (residuals). This last feature allows users to eventually explore different parameter selection rules.

### 3.3. Addressing the performance of *Raynals* with experimental DLS data

**Figure 4:**
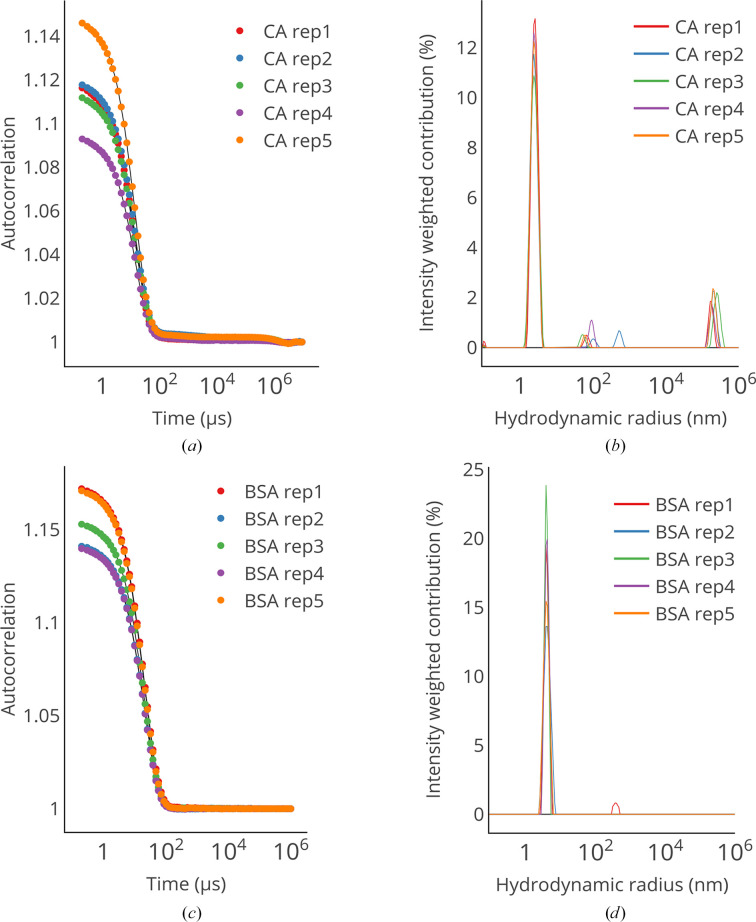
(*a*, *c*) Autocorrelation curves of carbonic anhydrase (CA) and bovine serum albumin (BSA). (*b*, *d*) The estimated relative contributions of each hydrodynamic radius for CA and BSA. The regularization parameters were determined using the *L*-curve criterion.

To assess the performance of *Raynals* on experimental data, we conducted DLS experiments using a plate reader (wavelength of 817 nm and detection angle of 150°) on two extensively characterized proteins: carbonic anhydrase (CA) and bovine serum albumin (BSA) (Fig. 4 ▸). The estimated *R*_h_ values for CA and BSA were 2.3 ± 0.06 and 3.9 ± 0.08 nm (mean ± standard deviation of the WHM), respectively, in complete agreement with previously reported values (Graewert *et al.*, 2020 ▸; Brownsey *et al.*, 2003 ▸; Jachimska *et al.*, 2008 ▸). For these measurements, we decided to collect the data following centrifugation. However, in other cases it could be beneficial to apply DLS to report on the levels of aggregates that are present in the sample before centrifugation (and/or filtering).

In addition, we acquired DLS curves from commercially available gold nanoparticles (GP) with a radius of 2.5, 10 or 20 nm. Since it was not possible to centrifuge the samples due to sedimentation of the GP, large dust particles present in the sample strongly contributed to the scattering signal and dominated the measurements for the 2.5 nm GP. For the 10 nm and 20 nm GP, even though the curves were noisy and presented more than one transition, the observed *R*_h_ values were 11 ± 1.3 and 19.5 ± 2.8 nm, respectively, in agreement with the expected values (Supplementary Fig. S6).

**Figure 5:**
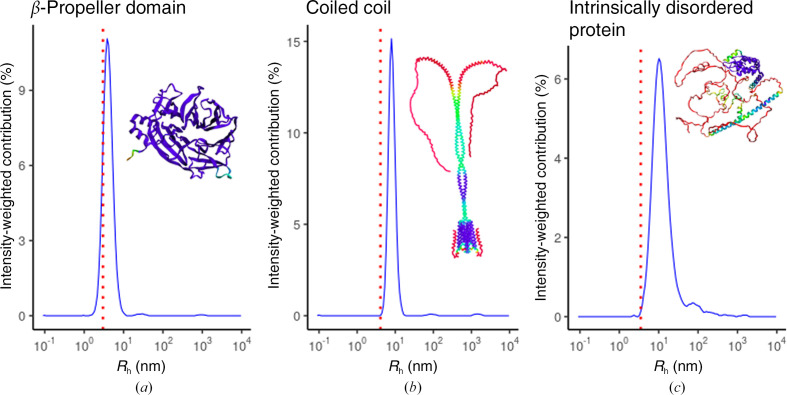
DLS measurements and *AlphaFold* model predictions of in-house samples. The 30 acquisitions were fitted separately and the estimated intensity values were then averaged. Hydrodynamic radius (*R*_h_) distribution (blue lines) of the β-propeller domain (*a*), coiled-coil polypeptide (*b*) and IDP (*c*). Red dashed lines represent the expected *R*_h_ based on the molecular weight and a globular model. *AlphaFold* models are coloured by pIDDT values (very low, red; low, yellow; OK, green; confident, light blue; very high, violet).

In addition, we tested the performance of *Raynals* analysis using a second DLS device with a cuvette holder (wavelength of 658 nm and detection angle of 90°) on in-house-produced proteins (Fig. 5 ▸). We used a β-propeller domain (BPD), a polypeptide that adopts a coiled-coil structure (CC) and an intrinsically disordered protein (IDP). The mean WHM from 30 acquisitions was 3.8 ± 0.2, 7.9 ± 0.4 and 10.1 ± 0.6 nm, respectively. It is important to remember that *R*_h_ is derived from the diffusion coefficient and requires the assumption of spherical hard particles (Stokes–Einstein relation, equation 6).

BPD is a monomeric globular protein (ter Haar *et al.*, 1998 ▸) of 40 kDa. CC has a molecular weight of 53 kDa and is a stable dimer (Yang *et al.*, 1999 ▸) and IDP has a molecular weight of 65 kDa. Comparison of the approximated radii for each of these proteins highlights the versatility and limitations of using DLS to assess, for example, the oligomeric state of the proteins; the globular BPD is not dissimilar in molecular weight to IDP, although due to the disordered region the calculated *R*_h_ is almost 2.5 times greater. If IDP were assessed as a globular protein, the approximate molecular weight would differ by at least two orders of magnitude from the correct value. CC has a radius of two times the value of BPD although it is only twice the weight. For these reasons, absolute *R*_h_ values should not be used to analyse oligomerization.

### 3.4. Comparison of *Raynals* with other DLS analysis software

*Raynals* can be compared with other DLS software such as *CONTIN* (Provencher, 1982 ▸), *SEDFIT* (Brown *et al.*, 2007 ▸) and the commercial software *DYNAMICS* (https://www.wyatt.com/products/software/dynamics.html) (Table 3 ▸). While these three programs require installation under a particular operating system, *Raynals* is available online and can be executed from a browser, which facilitates access by potential users independent of the performance of their computer. One of the strong points of *CONTIN* is the availability of the code as open source. However, it is written in Fortran, which limits the possibility for users to adapt it for their personal purposes (for example changing the regularization matrix). The code for fitting DLS data from *Raynals* is available on GitHub (https://github.com/osvalB/dynamicLightScatteringAnalysis) and should be easier to modify, as it is written in Python.

The four programs use a regularization approach for data fitting, but *CONTIN* and *SEDFIT* only allow a single curve to be fitted at a time. The increase in the availability of plate-based DLS, which allows the screening of multiple conditions, creates the need for data-comparison tools. Both *Raynals* and *DYNAMICS* stand out in their ability to analyse and plot multiple curves at the same time. *Raynals* is designed with advanced features that allow the comparison of peaks in defined regions of the distribution. Interestingly, analysis of *R*_h_ is performed by automatic peak detection and this is the value that is reported, facilitating analysis of the region of interest in the distribution. This feature permits the easy comparison of *R*_h_ while the user is experimentally screening for different conditions (for example different buffers during sample optimization). In contrast, the previously existing software retrieves *R*_h_ as an average which is more prone to be disturbed by small events or peaks.

It is fair to mention that each software also has unique features. *DYNAMICS*, for instance, can model concentration-dependent size changes. This is an interesting addition that would allow users to estimate the critical micelle concentrations (CMCs) of detergents, for example. Meanwhile, *CONTIN* is capable of performing global analysis where an external parameter is varied (for example, the angle of detection; Provencher & Štêpánek, 1996 ▸) and *SEDFIT* allows the use of prior probabilities before fitting the data (for example, the peak location).

*Raynals* has been designed as a powerful tool to address the quality control of biological samples during sample preparation and optimization. It possesses useful features for experiment planning and training. Simulation of autocorrelation curves can be performed using the expected *R*_h_ from one or many populations of particles. DLS is a simple instrument to use, but the data can easily be misinterpreted. The simulation tools available in *Raynals* allow an understanding of the limitations of the measurement and its resolution, help in the detection of aggregates and show the influence of large particles over the recorded signal. Additionally, *Raynals* provides flexibility in the way that the data are presented: the distribution of decay rates (or diffusion coefficients) can be visualized as histograms, density plots or grayscale bar plots in publication-quality format.

## 4. Conclusions

DLS is a widely used technique to obtain information about the size and dispersity of macromolecular samples. The fitting of the acquired data entails the solution of a nonlinear inverse problem, for which the Tikhonov–Phillips regularized inversion has been suggested as a promising approach. Our research, based on both simulations and experimental data, including two DLS instruments, diverse proteins and gold nanoparticles, supports the idea of this method producing reliable results. We look forward to receiving feedback from the scientific community and expanding our software to include more complex analyses such as multi-angle fitting and temperature ramps. Furthermore, *Raynals* offers a simulation panel that can help users to gain a deeper understanding of the challenges of the technique.

## 5. Figures

Figs. 1 ▸ and 2 ▸ were generated with *Inkscape* (https://www.inkscape.org). Figs. 3 ▸ and 5 ▸ were generated with the *R* package *ggplot*2 (Wickham, 2016 ▸). The plots in Fig. 4 ▸ were directly exported from *Raynals* and combined with *Inkscape*.

## 6. Data and code availability

The experimentally and artificially generated DLS data together with the *R* scripts used to produce Fig. 3 ▸, Fig. 5 ▸ and Supplementary Figs. S1–S4 can be downloaded at Zenodo (https://doi.org/10.5281/zenodo.7856850). The Python code used to fit the DLS data is available at https://github.com/osvalB/dynamicLightScatteringAnalysis.

## Supplementary Material

Supplementary Figures and protein sequences. DOI: 10.1107/S2059798323004862/vo5014sup1.pdf

Dynamic light scattering datasets used to assess the Raynals software: https://doi.org/10.5281/zenodo.7856850

## Figures and Tables

**Table 1:** Experimental setup for the DLS experiments CA, BSA, BPD, IDP and CC were measured at concentrations of 1, 1, 5, 2 and 3 mg ml^−1^, respectively. GP (10 nm) and GP (20 nm) were measured using 1:10 and 1:100 dilutions from the stock solution.

Experiment	Technical replicates	Acquisition time (s)	No. of acquisitions
CA	5	10	20†
BSA	5	1	20†
GP	2	1	6†
BPD	1	5	30
IDP	1	5	30
CC	1	5	30

† Each final DLS curve represents the average of the total number of acquisitions.

**Table 2:** Spearman’s correlation between the estimated and true standard deviation for four groups of samples with a constant mean hydrodynamic radius (*R*_h_) and different standard deviations (11 subsamples) Correlation plots are shown in Supplementary Fig. S2.

	α				
*R*_h_	0.0001	0.001	0.01	0.1	1
2 nm	0.6	0.6	0.7	0.6	0.5
6 nm	0.7	0.8	0.9	0.9	0.9
18 nm	0.8	0.9	0.9	1.0	1.0
54 nm	0.6	0.7	0.8	0.9	0.9

**Table 3:** Comparison of software for the analysis of DLS data

Software	Online	Open source	Open access	Analysis of multiple curves
*Raynals*	Yes	Partially†	Yes	Yes
*CONTIN*	No	Yes	Yes	No
*SEDFIT*	No	No	Yes	No
*DYNAMICS*	No	No	No	Yes

† We provide the code for the data-analysis step (the code for the user interface is not available).
